# Factors Associated With Dietary Quality During Initial and Later Stages of the COVID-19 Pandemic in Mexico

**DOI:** 10.3389/fnut.2021.758661

**Published:** 2021-12-15

**Authors:** Carolina Batis, Laura Irizarry, Analí Castellanos-Gutiérrez, Tania C. Aburto, Sonia Rodríguez-Ramírez, Dalia Stern, Carla Mejía, Anabelle Bonvecchio

**Affiliations:** ^1^National Council for Science and Technology (CONACYT) – Nutrition and Health Research Center, National Institute of Public Health, Cuernavaca, Mexico; ^2^Nutrition Unit, World Food Programme Regional Bureau for Latin America and Caribbean, Panama City, Panama; ^3^Nutrition and Health Research Center, National Institute of Public Health, Cuernavaca, Mexico; ^4^National Council for Science and Technology (CONACYT) – Population Health Research Center, National Institute of Public Health, Cuernavaca, Mexico

**Keywords:** COVID-19, lockdown, diet quality, Mexico, adults

## Abstract

**Background:** The COVID-19 pandemic disrupted the global economy and modified lifestyles. The aim of our study was to identify factors associated with dietary quality, and their frequency, in Mexican adults at the initial and later stages of the pandemic.

**Methods:** Two online surveys were conducted between June and July 2020 (*n* = 3,131) and between November and December 2020 (*n* = 1,703 including non-participants from 1st round). A diet quality score was estimated using a short instrument to measure the consumption of several healthy/unhealthy food items. Linear regression models were used to identify the association between pandemic related factors and the diet quality score, adjusted by sociodemographic characteristics. The 2nd round was weighted to represent the 1st round.

**Results:** During the 1st and 2nd rounds only ~12% of the sample perceived that their intake of healthy food decreased, relative to before the pandemic; ~20% perceived that their intake of unhealthy foods increased. Diet quality remained similar between the 1st and 2nd round. The following factors were negatively associated with diet quality: Eating food prepared away-from-home; going out to work ≥4 times/week; decreased time for food preparation; decreased interest in eating healthy; eating more due to anxiety, depression, or boredom; food insecurity; and stockpiling junk food. Purchasing food using a mixed modality of both in-store and home delivery was positively associated with diet quality. With the exception of eating more due to anxiety (reported by 47% of participants), all these factors were reported by a minority of participants during the first round (≤15%). During the 2nd round, there was an increase in the frequency of participants who reported eating food prepared away-from-home, going out to work ≥4 times/week, having less time to prepare food, being more interested in eating healthfully, and a decrease in participants eating more due to anxiety, depression or boredom, or stockpiling junk food.

**Conclusions:** Most participants perceived that their dietary intake improved during both initial and later stages of the pandemic. This might be related to factors associated with higher dietary quality, such as not going out to work, eating homemade food, and online grocery shopping.

## Introduction

Mexico documented its first case of COVID-19 on February 27th, 2020. In little over two months, close to 20,000 confirmed cases were registered (1). A year later, over two million cases and 228,000 deaths had been officially documented in the country (2). In an effort to slow the spread of COVID-19 in the country, a national public health emergency was declared in March 2020. While mandatory lockdowns or curfews were never in place, federal government efforts promoted a stay-at-home campaign (“Quédate en Casa”) and encouraged social distancing measures (3). Nationwide, all educational institutions remained closed for over 15 months, some re- opening on June 7th, 2021. From mid-march to the end of May 2020, only essential economic activities were permitted, and from June 2020 onwards, a state-specific traffic light system was established to indicate the level of economic activities permitted, as well as the use of public spaces according to the risk of infection by SARS-COV-2.

Concerns have been raised about the impact of COVID-19 on the nutritional status of individuals (4, 5). The Mexican population was already nutritionally vulnerable prior to the start of the COVID-19 pandemic. Over 55% of Mexican households have some degree of food insecurity (6). Overweight and obesity are widespread, affecting 70% of Mexican adults, close to 40% of adolescents, and 35% of children (7, 8). Undernutrition and micronutrient deficiencies are also enduring public health challenges among segments of the population (9). The elevated consumption of foods that are high in saturated fat and/or added sugar and low nutrient density (discretionary foods) and sugar-sweetened beverages, coupled with inadequate consumption of essential foods such as fruits, vegetables and legumes before the pandemic, are known to have contributed to the double burden of malnutrition (10, 11). The economic implications of the pandemic, alongside those resulting from confinement and social distancing measures predictably influenced access to food, food security, purchasing behaviors, dietary patterns, and general lifestyle (12–14).

Understanding the impact of the COVID-19 pandemic on nutrition-related behaviors—in the short and long term—is imperative. Surveys conducted to date around the world have shown mixed findings, with some segments of the population reporting improvement in dietary habits while others reporting the opposite (even within the same survey) (15–20). In Mexico and Latin America, most studies report either no change or an improvement in dietary habits (19–22). Results from the Brazilian NutriNet cohort comparing food intake in adults before and during the confinement period show an overall increase in the intake of fruits, vegetables, and legumes, and no significant change in the intake of ultra-processed foods (20). In a cross-sectional online survey disseminated trough social media during the confinement period in several Ibero-American countries, it was found that in Argentina, Brazil, Mexico, and Peru, most participants reported no change in their dietary habits compared to before confinement, and among those who changed their diet, the majority of participants from all countries except for Peru did so toward a healthier diet (19). Other online surveys in Mexico report a perceived increase in diet quality during quarantine (21) or a higher percentage of participants that report having a healthy diet during confinement compared to before confinement, but also 30 to 50% that report increasing their intake of sweets, desserts, sugar-sweetened beverages, and/or junk food (22). Yet, in low- and middle-income countries, including Mexico, an increase in food insecurity has been reported, as well as a decrease in diet diversity, particularly among those from low socioeconomic status (SES) (13, 23). These discrepancies could result from the interplay between individual characteristics and the specific context or life situation faced during the pandemic. Hence, assessing the relation between factors related to the pandemic and dietary quality, and in which segments of the population these factors were more frequent, can assist in better understanding the impact of the pandemic on dietary quality.

We conducted two online surveys among Mexican adults at initial and later stages of the pandemic. Our aim was to identify self-perceived changes in dietary habits and to evaluate the association between pandemic-related factors (e.g., home confinement, grocery shopping mode, consumption of food prepared away-from-home, emotional eating, food insecurity, changes in income, free time, time for cooking, interest in healthy eating, etc.) and diet quality. In addition, we identified the frequency of these pandemic-related factors during initial and late stages of the pandemic (1st and 2nd round) and their distribution according to sociodemographic and individual characteristics.

## Methods

### Study Population

We conducted two online surveys among Mexican adults, the 1st round between June 24th and July 27th, 2020, and the 2nd round between November 12th and December 16th, 2020. The first survey was conducted when the pandemic was in its initial stages and the second almost a half year later when the novelty of the pandemic had decreased and there were less restrictions. At the time of the 1st survey, mobility nationwide had been reduced by 40–70% and by the time the 2nd survey was conducted, mobility was down by 10–45% (24). Both surveys included the same questionnaire. Inclusion criteria were being age 18 or older and living in Mexico at the time of the survey. The 1st online survey round was disseminated through the institutional social media accounts of the Mexican National Institute of Public Health (INSP) and the World Food Programme (WFP), partner institutions, civil society, and the authors' personal social media networks. Paid advertisements on Facebook were also used to enhance the reach and diversity of the sample. The same diffusion strategy was used for the 2nd round and, in addition, email invitations were sent to 1st round participants who voluntarily provided an email for follow-up. The 2nd round was open to subjects that did not participate in the 1st round. Informed consent was obtained from each participant prior to starting the survey. The survey protocol was reviewed and approved by the Research and Ethics Committees of the INSP.

Surveys were collected through MODA (Mobile Operational Data Acquisition), the web-based platform used by the World Food Programme for data collection. The instrument was pilot tested before data collection, included 49 questions and took 10 to 15 min to complete. Participants were required to answer all questions to submit the survey. A total of 3,131 adults participated in the 1st round and 1,703 in the 2nd round (from which 766 reported participating in the 1st round and 522 were confirmed to have participated in both rounds by matching their email addresses).

### Questionnaire Sections

#### Sociodemographic and Individual Characteristics

Sociodemographic variables collected included sex, age, marital status, geographical location (state/municipality), occupation before and after the start of the pandemic, head of the household education level, household composition, and government support benefits. SES was assessed using the Mexican Association of Market Research Agencies and Public Opinion Index (25). This index classifies households into seven strata (from higher to lower: A/B, C+, C, C–, D+, D, E) based on six variables (number of bathrooms, rooms, vehicles, household members working, internet connection, and head of household education level). Employing this widely used index allowed us to compare the SES of our sample to that of the general Mexican population. Additionally, an individual characteristic regarding the importance attributed to health and nutrition was collected by asking the participants how often they usually choose foods according to their healthfulness (hereafter referred to as *healthy food consciousness*).

#### Diet Quality

To assess diet quality, participants were asked to recall all foods consumed the previous day and select them from a list of 31 food items. Quantities consumed were not measured. Food items were grouped into seven food categories (vegetables, fruits, animal and plant sources of protein, cereals, sweets, snacks, and ready-to-eat foods and beverages). Each category included the option “I did not consume any of the foods listed above,” intended as a prompt for the participant, but also to ensure that he/she selected at least one option from each category, given that an answer to all questions was required (Supplementary Table 1). To conform the diet quality score, points were assigned for the intake of each healthy item or the non-intake of each unhealthy item. Healthy items included fruits, vegetables, legumes, nuts and seeds, poultry, fish, eggs, and unsweetened grains. Unhealthy items included processed meat, sweets, snacks, ready-to-eat meals, and sugary beverages. The maximum score was 100 points (Supplementary Table 2). Further details about the development and performance of this instrument and the diet quality score in relation to 24-hr dietary recall data from the National Health and Nutrition Survey are described in the Supplementary Material section. We found that this score had a small correlation with micronutrient adequacy, and in the case of fiber, saturated fat, and added sugar it had a moderate correlation that was comparable to those found with more intricate diet quality indicators, such as the Alternate Healthy Eating Index-2010 or the energy share of ultra-processed foods (Supplementary Table 3) (26, 27).

#### Perceived Changes in Diet, Physical Activity, and Body Weight

Perceived changes in diet were assessed with the following questions: “Since the start of the pandemic, has your intake of healthy foods such as fruits, vegetables, whole grains, legumes, or plain water changed?”, and “Since the start of the pandemic, has your intake of unhealthy foods such as chips, sodas, cookies, or pastries changed?”, with the response options: decreased, increased, or unchanged. Participants were also asked about perceived changes in physical activity patterns and body weight (same response options). One of the response options perceived changes in body weight was being pregnant or in post-partum.

#### Pandemic-Related Factors

We were interested in identifying factors that were potentially affected or modified since the onset of the pandemic and that could, in turn, affect dietary intake (we refer hereafter to these factors as *pandemic-related factors)*. These factors included the level of home confinement during the previous 2-weeks; the consumption of food prepared away-from-home the previous day; shopping modality from grocery stores and traditional or street markets (*tianguis*) during the previous 2-weeks; household income changes since the start of the pandemic; perceived changes in free time; perceived time spent cooking; perceived interest in eating healthy; eating more due to anxiety, depression or boredom; food insecurity in the previous week; and food stockpiling or purchasing more than usual due to fear of scarcity. All prior questions referred to the time of the survey or to the perceived change from before the pandemic to the time of the survey. Only the shopping modality-related questions included additional questions regarding shopping habits before the onset of the pandemic.

An additional pandemic-related factor was the state-specific restriction level according to the traffic light system of epidemiological risk, which was obtained for each participant based on the date they answered the survey and their state of residence. The traffic light system considers four stages (red: maximum risk, only essential economic activities allowed; orange: high risk, non-essential economic activities at 30% capacity; yellow: moderate risk, only indoor public spaces at reduced capacity; and green: low risk, all activities functioning normally with basic prevention measures).

### Statistical Analysis

For each survey round, descriptive statistics were calculated to show the distribution of sociodemographic variables, food shopping modality (e.g., in-store vs. home delivery) before the pandemic and at the time of the survey, and perceived changes in diet, physical activity, and body weight. We estimated the mean diet quality score by sociodemographic and individual characteristics and ran unadjusted linear regression models to evaluate these associations.

To evaluate the association between pandemic-related factors and diet quality, we used linear regression models with diet quality score as the dependent variable and the pandemic-related factors as the independent variables. For each pandemic-related factor, we ran two models: (1) adjusted by covariates (sociodemographic and individual characteristics), and (2) additionally adjusted by all other pandemic-related factors. Our model of interest was the first one as we did not consider pandemic-related factors as confounders of each other. However, because it might be of interest to identify its independent association with diet quality, we included the second model.

**Model 1**


$$\begin{array}{l} Y\left(Diet\;quality\;score\right)\;=\;a_{0}\;+\beta 1\left(pandemic\;factor\;1\right)\\ +\;\gamma Covariates+e \end{array}$$

**Model 2**


$$\begin{array}{l} Y\left(Diet\;quality\;score\right)\;=\;a_{0}\;+\beta 1\left(pandemic\;factor\;1\right)\\ +\;\beta 2\left(pandemic\;factor\;2\right)\\ +\;\beta n\left(pandemic\;factor\;n\right)+\;\gamma Covariates+e \end{array}$$

We identified the frequency with which pandemic-related factors that were positively or negatively associated with diet quality were present in our sample. We also identified if the frequency differed by sociodemographic and individual characteristics such as sex, age, SES, and healthy food consciousness with a chi-square test.

For the analysis of the 2nd survey round, participants were weighted to be representative of the participants of the first round. Inverse Probability Weights (IPW) are a way to deal with missing data, account for lost to follow-up, and achieve comparability across rounds of data collection in longitudinal studies (28–30). We estimated IPW with the inverse of the probability of being in the second round (vs. the first) conditional on all sociodemographic variables. To estimate the weights, we ran a logistic regression with the survey round regressed on the sociodemographic variables and we obtained the estimated predicted probabilities. Instead of including 1 in the numerator when estimating the inverse, we stabilized the weights by including in the numerator the probability of being in the second round (not conditioning on any variable) (28).


$$\begin{array}{l} IPW=\frac{\text{P}\left(2\text{nd round}\right)\;}{\text{P}\left(2\text{nd round }|\;\text{Covariates}\right)} \end{array}$$

Our primary analysis was with all participants of the 2nd round (*n* = 1,703), but in the Supplementary Material we also present results with the subsample that reported participating in 1st round (*n* = 766), and with the subsample in which participation in the 1st round was confirmed and linked to an email (*n* = 522). The analysis was conducted in STATA 15 (StataCorp, College Station, TX). For all analyses, we used a *p*-value < 0.05 to consider results significant. Furthermore, weighted estimations were obtained with the “svy” STATA module.

## Results

### Sample Characteristics

The 1st round of the survey was predominantly completed by women (76%) and the mean age of participants was 41. Participants from all states of Mexico were surveyed, but the majority were from Mexico City Metropolitan Area (40%). Almost all participants (92%) lived in highly urbanized municipalities (≥100,000 habitants). Overall, the education level of the head of household was high (43% had a bachelor's degree and 39% a graduate degree), and the majority were from high and middle SES. The majority (80%) reported choosing foods based on their healthiness always or almost always (healthy food consciousness). For the 2nd round, there were more females, young (18–30 years) and single participants compared to 1st round. Weighting the estimations of the 2nd round achieved comparability in the distribution between the two samples. The mean diet quality score was 64.2 (out of 100 possible points) in the 1st round and 64.4 in the 2nd round (Table 1). Diet quality was higher among individuals with the following characteristics: Female, older, married or with a partner, higher education, not studying or working, middle-high SES, receiving financial aid, with no children living in the same household, and more healthy food conscious (Supplementary Table 4).

**Table 1:** Sociodemographic and individual characteristics and mean diet quality score.

	**First survey round (Jun–Jul 2020)(*****n*** **=** **3,131)**		**Second survey round (Nov–Dec 2020)** **(*****n*** **=** **1,703)**		
	**n**	**%**	**n**	**Unweighted %**	**Weighted^**a**^ %**
Sex					
Female	2,367	75.6	1,366	80.2	77.2
Male	764	24.4	337	19.8	22.9
Age, %					
18–30 years	762	24.3	595	34.9	27.8
31–40 years	987	31.5	514	30.2	31.0
41–50 years	637	20.3	297	17.4	19.8
51–60 years	429	13.7	175	10.3	12.4
>60 years	316	10.1	122	7.2	9.0
Marital Status					
Single	1,096	35.0	754	44.3	37.9
Married or with partner	1,732	55.3	826	48.5	53.2
Divorced/separated/widowed	303	9.7	123	7.2	8.9
Head of the household highest education level					
Secondary school or less	231	7.4	175	10.3	8.1
High school	337	10.8	165	9.7	10.3
Bachelor degree	1,344	42.9	715	42.0	42.5
Graduate degree	1,219	38.9	648	38.0	39.1
Main occupation before the pandemic					
Student or working	2,275	72.7	1,140	66.9	71.0
Other	856	27.3	563	33.1	29.0
Socioeconomic status					
High (A/B)	647	20.7	398	23.4	21.4
Middle high (C+)	1,214	38.8	646	37.9	38.6
Middle low (C and C–)	1,131	36.1	593	34.8	35.8
Low (D+ and D)	139	4.4	66	3.9	4.2
Beneficiary of social programs					
None	2,930	93.6	1,603	94.1	93.5
Financial aid	111	3.6	50	2.9	3.5
Other	90	2.9	50	2.9	3.0
Geographical region					
South	593	18.9	303	17.8	18.4
Center	665	21.2	432	25.4	22.5
North	367	11.7	178	10.5	11.1
Mexico City Metropolitan Area	1,245	39.8	690	40.5	40.3
Guadalajara Metropolitan Area	261	8.3	100	5.9	7.8
Municipality population size					
≥1,00,000 habs.	2,865	91.5	1,509	88.6	90.7
<1,00,000 habs.	266	8.5	194	11.4	9.3
Household with children (<18 years)					
No	1,887	60.3	1,035	60.8	60.5
Yes	1,244	39.7	668	39.2	39.5
Healthy food consciousness					
Always	868	27.7	441	25.9	27.2
Almost always	1,666	53.2	944	55.4	54.2
Sometimes or never	597	19.1	318	18.7	19.6
	n	Mean (95% CI)	n	Unweighted mean (95% CI)	Weighted^a^ mean (95% CI)
Diet quality score	3,131	64.1 (63.8, 64.5)	1,703	64.3 (63.7, 64.8)	64.4 (63.9, 64.9)

a *Weighted to maintain the distribution of sociodemographic variables of 1st round's participants*.

### Perceived Changes in Diet, Physical Activity, and Weight During the COVID-19 Pandemic

**Figure 1:**
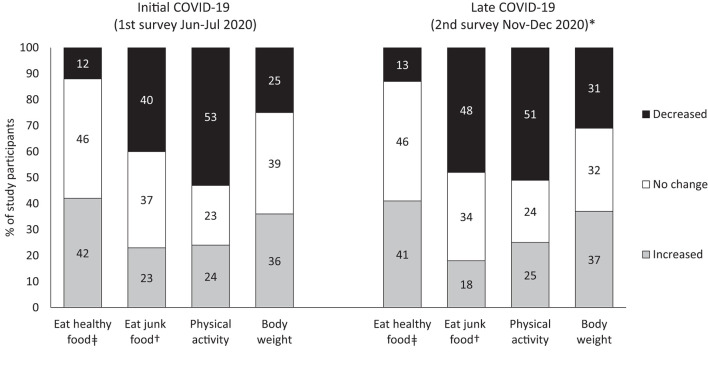
Perceived changes in diet, physical activity and body weight, between pre-pandemic to initial and later stages of COVID-19 pandemic (1st and 2nd survey round). *Weighted to maintain the distribution of sociodemographic variables of 1st round's participants. ^‡^Fruit, vegetables, whole grains, legumes, plain water. †Chips, sodas, cookies, pastries.

During the 1st round, 42% of the study sample perceived that their intake of healthy foods increased during lockdown and 40% perceived that their intake of unhealthy foods decreased. Half of the sample perceived that the time they spent on physical activity decreased. Excluding pregnant or post-partum participants, 39% of respondents perceived unchanged weight, 36% increased weight, and 25% perceived a decrease in weight. During the 2nd survey round, results (weighted to maintain comparability with 1st round) were similar, except that the proportion that perceived that their intake of unhealthy foods decreased during lockdown reached 48% (Figure 1).

### Association Between Pandemic-Related Factors and Diet Quality

In Table 2, we present the association between the pandemic-related factors and diet quality during the 1st survey round. Adjusted by covariates (Model 1), the following pandemic-related factors had a statistically significant negative association with diet quality: Eating food prepared away-from-home compared to not doing so [−2.6 (95% CI: −3.6, −1.6) from restaurants and −8.1 (−10.0, −6.1) from street vendors]; going out to work ≥4 times/week compared to going out for motives other than work [−2.1 (−3.1, −1.1)]; decreased interest in eating healthy during lockdown compared to no change [−4.4 (−5.8, −3.0)]; eating more due to anxiety, depression or boredom compared to not doing so [−2.4 (−3.1, −1.7)]; skipping meals, eating less, or not eating in an entire day due to economic constraints (food insecurity) compared to not having difficulty [−3.5 (−4.9, −2.1)]; and stockpiling junk food compared to not stockpiling any kind of food [−4.9 (−6.3, −3.5)]. In comparison to no change during lockdown, decreased time for cooking was negatively associated [−2.9 (−4.9, −1.0)] and increased time was positively associated with diet quality [1.0 (0.1, 2.0)]. Finally, purchasing food with a combination of in-store and home delivery was associated with increased diet quality for both traditional/street markets [3.2 (0.7, 5.7)] and grocery stores [2.0 (0.7, 3.3)]. Further adjusting by all other pandemic-related factors (Model 2) weakened the association in most cases. Results were similar for the 2nd survey round (Supplementary Table 5).

**Table 2:** Association between pandemic related-factors and diet quality score during the 1st survey round.

	**Model 1**	**Model 2**
Food prepared away-from-home the day before		
None	0 (ref)	0 (ref)
Restaurant (includes take-out and delivery)	−2.6 (−3.6, −1.6)	−2.2 (−3.2, −1.3)
Street vendors	−8.1 (−10, −6.1)	−7.5 (−9.5, −5.6)
Traditional or street market purchases, now		
In-store	0 (ref)	0 (ref)
In-store and home delivery	3.2 (0.7, 5.7)	3.1 (0.7, 5.5)
Home delivery	1.1 (0, 2.3)	1.0 (−0.2, 2.1)
None	−0.7 (−1.5, 0.1)	−0.9 (−1.7, −0.1)
Grocery store purchases, now		
In-store	0 (ref)	0 (ref)
In-store and home delivery	2.0 (0.7, 3.3)	2.0 (0.7, 3.3)
Home delivery	0.2 (−0.8, 1.1)	0.0 (−1.0, 0.9)
None	0.8 (−0.2, 1.8)	0.4 (−0.6, 1.4)
Level of home confinement		
Going out for motives other than work	0 (ref)	0 (ref)
Not leaving the home	−0.7 (−2, 0.7)	−0.8 (−2.1, 0.6)
Going out to work ≤3 times/week	−0.7 (−1.6, 0.3)	−0.7 (−1.6, 0.2)
Going out to work ≥4 times/week	−2.1 (−3.1, −1.1)	−1.6 (−2.6, −0.6)
Income changes		
No change	0 (ref)	0 (ref)
Increased	0.7 (−1.7, 3.2)	1.7 (−0.7, 4.1)
Decreased somewhat	−0.1 (−1, 0.7)	−0.2 (−1, 0.6)
Decreased a lot	0.2 (−0.7, 1.1)	0.9 (0, 1.9)
Perceived change in free time		
No change	0 (ref)	0 (ref)
Decreased	0.4 (−0.6, 1.5)	0.8 (−0.2, 1.8)
Increased	0.8 (−0.1, 1.7)	0.7 (−0.2, 1.6)
Perceived change in time for cooking		
No change	0 (ref)	0 (ref)
Decreased	−2.9 (−4.9, −1)	−0.9 (−2.8, 1)
Increased	1 (0.1, 2)	0.9 (0, 1.8)
Food is prepared by others	−0.9 (−2, 0.2)	−0.6 (1.7, 0.4)
Perceived change in interest in eating healthy		
No change	0 (ref)	0 (ref)
Decreased	−4.4 (−5.8, −3)	−3.1 (−4.5, −1.7)
Increased	1 (0.3, 1.7)	0.7 (0, 1.4)
Eating more due to anxiety, depression or boredom		
No	0 (ref)	0 (ref)
Yes	−2.4 (−3.1, −1.7)	−1.7 (−2.4, −1)
Food insecurity^a^		
No difficulty	0 (ref)	0 (ref)
Cheaper foods or that I enjoy less	−0.7 (−1.7, 0.3)	−0.5 (−1.6, 0.5)
Skip meals, eat less, or do not eat in an entire day	−3.5 (−4.9, −2.1)	−3.3 (−4.8, −1.9)
Stockpiling food		
None	0 (ref)	0 (ref)
Only basic foods	0.6 (−0.1, 1.3)	0.5 (−0.2, 1.3)
Junk food	−4.9 (−6.3, −3.5)	−4.3 (−5.7, −2.9)
Restriction level		
Orange	0 (ref)	0 (ref)
Red	−0.3 (−1.0, 0.4)	−0.1 (−0.8, 0.6)

a *Dificulty eating enough due to economic constraints*.

*Model 1 adjusted by sex, age category, marital status, education level from head of household, main occupation before the pandemic, SES, beneficiary of social programs, geographic region, municipality population size, household with children, and healthy food consciousness*.

*Model 2 adjusted by covariates from Model 2, plus all the other pandemic-factors listed in this table*.

### Distribution of Pandemic-Related Factors

During the 1st survey round, most pandemic-related factors negatively associated with diet quality were present in a minority of the sample (≤15%). Only eating more due to anxiety, depression, or boredom was reported by 47% of the sample (Table 3). By the 2nd round (weighted to maintain comparability with 1st round), more participants reported consuming food prepared away-from-home, going out to work ≥4 times/week, and having decreased time for cooking; whereas less participants were using a combination of in-store and home delivery for their grocery shopping. However, more participants were interested in eating healthy, and less participants reported eating more due to anxiety, depression or boredom, and stockpiling junk food. By sociodemographic factors during the 1st survey round, in general we found that the factors negatively associated with dietary quality were more frequent (and the factors positively associated less frequent) among males, younger individuals, those with low SES, and those with low healthy food consciousness. Nonetheless, the opposite was true for eating more due to anxiety, depression or boredom, which was more frequent among females. In addition, having more time for cooking and increased interest in eating healthy was more frequent among younger people, while stockpiling junk food was more frequent among those with low SES (Table 3).

**Table 3:** Frequency of pandemic related factors negatively and positively associated with dietary quality by survey, sociodemographic, and individual characteristics.

		**1st Survey (Jun–Jul 2020)**								
	**Survey round**		**Sex**		**Age**		**Socioeconomic status**			**Healthy food** **consciousness** ^**a**^	
	**1st**	**2nd^**b**^**	**F**	**M**	**≤60**	**>60**	**High** **(A/B)**	**Middle** **(C+ / C-)**	**Low** **(D+ / D)**	**High**	**Low**
**Factors negatively associated with dietary quality**											
Eating from restaurant (includes take-out and delivery) the day before	15	21^*^	14	18^*^	16	11^*^	17	15	14	15	20^*^
Eating from street vendors the day before	4	5^*^	3	4	3	1^*^	3	3	4	3	7^*^
Going out to work ≥4 times/week	15	25^*^	14	20^*^	17	5^*^	16	15	24^*^	15	21^*^
Perceived decreased time for cooking	4	7^*^	4	2^*^	4	4	3	4	9^*^	4	5
Perceived decreased interest in eating healthy	7	7	8	6	8	4^*^	7	7	12	6	15^*^
Eating more due to anxiety, depression or boredom	47	42^*^	50	39^*^	49	28^*^	47	46	58^*^	45	61^*^
Food insecurity (skip meals, eat less, or do not eat in an entire day)^c^	7	5	7	6	7	2^*^	3	7	25^*^	6	9^*^
Stockpiling junk food	7	4^*^	7	5	7	3^*^	9	6	2^*^	5	11^*^
**Factors positively associated with dietary quality**											
Purchasing in-store and home delivery from traditional or street markets	2	2	2	3	2	1	1	2	4	2	1
Purchasing in-store and home delivery from grocery stores	8	6^*^	8	7	8	5	10	8	3^*^	8	6
Perceived increased time for cooking	52	44^*^	56	41^*^	53	47^*^	49	53	49	53	45^*^
Perceived increased interest in eating healthy	52	57^*^	51	55^*^	53	41^*^	49	52	48	51	47^*^

a *High: always or almost always choose foods according to their healthfulness; Low: sometimes or never*.

b *Weighted to maintain the distribution of sociodemographic variables of first round's participants*.

c *Dificulty eating enough due to economic constraints*.

* *p value < 0.05*.

**Figure 2:**
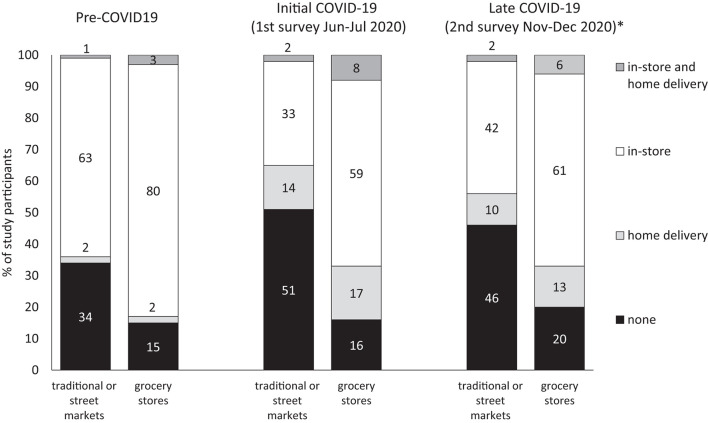
Food shopping place and modality pre-COVID 19 and during initial and later stages of COVID-19 pandemic (1st and 2nd survey round). *Weighted to maintain the distribution of sociodemographic variables of 1st round's participants.

Changes in self-reported food purchasing patterns were observed from the time before the pandemic to the time of the survey. During the initial stages of the pandemic, for grocery stores purchases, 20% of participants switched from in-store-only to home-delivery options, maintaining the total proportion purchasing from grocery stores at ~85%. For traditional or street markets purchases, 13% switched to home-delivery options, but the total proportion purchasing from these places decreased from 66 to 49%. During the 2nd survey round (weighted to maintain comparability with 1st round), participants started to return to pre-COVID-19 patterns (Figure 2).

## Discussion

In these online surveys of Mexican adults carried out during initial (Jun–Jul) and later (Nov–Dec) stages of the COVID-19 pandemic, we found that the majority of the sample perceived that their dietary habits either did not change or improved. Many pandemic-related factors were associated with dietary quality. For instance, eating food prepared away-from-home, going out to work ≥4 times/week, decreased time for cooking, decreased interest in healthy eating, eating more due to anxiety, depression or boredom, food insecurity, and stockpiling junk food, were all negatively associated with diet quality. On the other hand, purchasing food using a combined modality of in-store and home delivery was positively associated with diet quality. The frequency in which the majority of these factors was reported was low, but its frequency was higher in some segments of the population, most notably among those who were younger, from low SES, and who had less healthy food consciousness (those that seldom chose their food based on their nutritional value). Furthermore, dietary quality remained similar between the 1st and 2nd survey rounds, likely because from initial to late lockdown, the frequency of several negative factors increased (consuming food prepared away-from-home, going out to work ≥4 times/week, and having less time to prepare food), but it was compensated by other changes. For instance, from initial to late lockdown, there was an increase in participants interested in eating healthy, and a decrease in participants who reported eating more due to anxiety, depression or boredom, and stockpiling junk food.

Worldwide, several studies have been conducted to understand the effect of the COVID-19 pandemic on dietary habits. Study designs range from online surveys such as our own, to pre-established cohorts with pre-pandemic and pandemic measures, and analysis of sales trends. For online surveys, sample size ranged from ~400 to ~3,500 participants. Studies from Latin America and Europe consistently reported that there was an increase (measured or self-perceived) in the intake of legumes (15, 20, 31). Many studies reported an increase in fruits and vegetables (20, 31–36), with few studies finding a decrease (17, 37). Results were mixed for snacks/sweets, with some studies reporting a decrease (15, 37), and others an increase (16, 31, 33, 38). Likewise, in most studies, including ours, a higher proportion of adults perceived that their diet quality improved rather than worsened (15, 17, 19, 35), with one study reporting the contrary (18). Interestingly, we found that 36% perceived they have gained weight during the pandemic. In three studies from the Middle East and Europe and in our study, the proportion of subjects who perceived weight gain during the pandemic was 30–48%, consistently higher than the proportion that perceived losing weight (14–25%) (15, 39, 40). These findings on weight change might be related to lower levels of physical activity and/or to eating more due to anxiety, depression, or boredom, which was reported by almost half of participants (47%). Overall, the evidence suggests that the pandemic had more positive than negative effects on the diets of those surveyed. However, this was not homogenously observed for all segments of the population. Individual characteristics as well as the particular experience and situation each individual faced during lockdown (e.g., presence or absence of pandemic-related factors) likely played a role in determining the overall effect of the lockdown on dietary intake.

Home confinement in itself and some closely related factors such as not eating food prepared away-from-home and spending more time cooking were positively associated with dietary quality in our study. Furthermore, the effect of home confinement was independent from the effect of eating food prepared away-from-home (e.g., mutually adjusted in Model 2). A previous analysis from the Mexican National Nutrition Survey found that the intake of sweetened beverages and discretionary junk food is lower at home (41). It is possible that homemade food is healthier, but also there might be less exposure to the widespread availability and opportunities to consume unhealthy food found away from home and in social interactions. Studies from other countries coincide in that during lockdown, home-cooking and the intake of homemade food increased, whereas the intake of fast-food or food from restaurants decreased (15, 31, 35, 37, 40, 42, 43). Also, other studies coincide in that spending more time at home was associated with healthier eating, particularly among those that used to have many meals/day away-from home (34, 38). Interestingly, we found that having more free time was not associated with dietary quality. This suggests that convenience might be a less important driver of unhealthy eating as opposed to the widespread availability of junk food outside the home.

Among our sample, food purchase patterns were also affected by the pandemic. We found that online food purchases increased; this was also reported in France, Brazil, and Morocco (17, 36, 43). Online food purchases can have a beneficial impact, since the shopper is not exposed to all of the store's marketing strategies, food cravings are reduced by not seeing the real food, and there is no immediate gratification (44). Remarkably, in our study, better diet quality was found among those that combined in-store with online shopping. One possibility is the bulk of their shopping was made online and that due to biosecurity measures, the in-store purchases were fast and limited to fresh produce (which are harder to order/select online). Thus, ensuring a supply of healthy produce while reducing the exposure to cravings and marketing strategies in the store. Furthermore, we found that despite the migration to home delivery options, the net proportion of participants that purchased from traditional and street markets decreased during the lockdown. Given that traditional/street markets sell mainly fresh produce, it was expected that not purchasing food from these places would have a negative effect on dietary intake. Several reasons such as markets closing, fear of becoming infected with the SARS-COV2 virus while shopping (due to the crowds, lack of safety measures or the need to interact with many buyers), or limited online or home delivery options, might explain the drop in the purchases from traditional/street markets (45). Also related to food purchases, fear of scarcity could lead to stockpiling of food. We found that few people (<15%) perceived food shortages during the 1st survey round (data not shown), yet 40% of the sample stockpiled food (33% basic food and 7% junk food). As anticipated, stockpiling junk food was associated with lower dietary quality. Interestingly, by the 2nd survey round, we found that all food purchases patterns mentioned above were returning to pre-COVID-19 levels.

The pandemic put a strain on the population's mental health, resulting from fear of becoming infected, uncertain situations, economic difficulties, and/or isolation. These negative feelings can trigger emotional eating as a coping mechanism (46). We found that almost half of the sample reported that they were eating more due to anxiety, depression, or boredom; but the proportion was much lower among males (38%) and older subjects (28%), and higher among those with lower SES (58%). Di Renzo et al. reported that in an Italian sample 61% had a depressed mood, 70% had anxious feelings, and 55% felt the need to increase food intake to feel better; and consistent with our study, females were more affected (46). On the flip side, given that this is a sanitary crisis, the population can become more aware of their health status and be motivated to improve their lifestyle habits. We found that half of the sample was more interested in healthy eating. Among Polish adolescents, health and weight control became more important determinants of food choice during lockdown (47).

Overall, we found that pandemic-related factors negatively associated with dietary quality were infrequent in our sample. This might explain why the majority perceived improvements or no changes in the healthiness of their intake during the lockdown. However, we found that there were important differences in the frequency of these negative factors by characteristics such as age, sex, and SES. Many negative factors were more frequent among low SES. For instance, food insecurity (skipping meals, eating less, or not eating in an entire day due to economic constraints) affected 4 to 7% of those in high and middle SES stratums (A/B to C-), yet it reached 26% among those with low SES (D+ and D). Consistent with our findings, according to another survey in Mexico conducted via telephone and with probabilistic sampling, food insecurity was experienced by 26% of those in the D+ stratum. However, this study captured even lower SES (E stratum) and it was reported that 50% experienced food insecurity (23). Also of interest was that most pandemic-related factors negatively associated with diet quality were more common among those that are less healthy food conscious. Likely, this is related to other associated sociodemographic factors, but it is also possible that being healthy food conscious can have a role in protecting individuals from factors that negatively impact dietary intake. For example, going out to work or not is not up to the individual, but it is also possible that individuals that are more concerned about health issues more actively seek remote working options; or food insecurity might be lower among individuals who, despite economic constraints, place a higher value on nutrition. More research is needed to understand if this is the case.

A key question is whether the pandemic will have a long-lasting effect on the factors observed, or if these will be reestablished once the pandemic subsides. In our 2nd round, many factors were changing in the expected direction. As mobility increased, it also increased the frequency of eating food prepared away-from-home, going out to work, and having less time for food preparation, while the frequency of stockpiling junk food decreased. Interestingly, we found that in comparison to initial (Jun–Jul) lockdown, during the later stage of the pandemic (Nov–Dec), the interest in eating healthy increased and eating more due to anxiety, depression or boredom decreased. The number of cases per day related to COVID-19 were 39% higher during the 2nd round (48). It is possible that by this time, the participants themselves or their close-ones had been infected, which raised awareness of participant's own health status. It could also be the case that there was less isolation and uncertainty surrounding the pandemic, which also decreased the urge to eat due anxiety, depression, or boredom. Future studies will be needed to identify the long-term effects.

Several limitations and strengths to this study must be considered. Because this was a web-based survey, respondents were predominantly from a high income and education background. Another limitation is that the reliability and the level of detail of the data obtained through online surveys are much lower relative to off-line survey methods (49). A strength of our survey is that it went beyond the measurement of current diet quality and self-perceived changes. We also identified a range of pandemic-related factors, which allowed for a better understanding of the drivers of dietary quality during this challenging time. Another strength was the collection of two survey rounds to identify differences over time. Follow-up of the same participants was very limited, but weighing the analysis was useful to obtain more comparable samples. In Supplementary Table 5 we present results with the subsample in which participation in both surveys was confirmed and results were similar.

The COVID-19 pandemic presents an unprecedented challenge to individuals and society. Amidst the negative impact, the abrupt disruptions in lifestyle can come with certain positive effects for the high and middle SES population. The majority of the sample perceived that their dietary intake either improved or remained unchanged both at initial and later stages of the pandemic. Some factors associated with better diet quality were the home confinement, not consuming food prepared away-from-home, having more time for cooking, purchasing food both in-store and home delivery, and an increased interest in eating healthy. Nonetheless, the pandemic could have also exacerbated negative factors such as eating more due to anxiety, depression or boredom, food insecurity, and the stockpiling of junk food. Hence, a segment of the sample perceived that their dietary intake was unhealthier since the start of the pandemic. Studies like ours are relevant for understanding how the pandemic and other day-to-day factors affected by the pandemic could influence dietary quality. The pandemic might provide new ways of approaching and prioritizing food intake in the long-run. We found that as the pandemic went on, home confinement and home-prepared meals started to decrease but other factors such as the interest in eating healthy increased. Future studies will be needed to understand the long-term impact of the pandemic, if any, on the population's dietary quality.

## Data Availability Statement

The raw data supporting the conclusions of this article will be made available by the authors, without undue reservation.

## Ethics Statement

The studies involving human participants were reviewed and approved by Research and Ethics Committees of the National Institute of Public Health (INSP). The patients/participants provided their written informed consent to participate in this study.

## Author Contributions

CB, LI, and AB conceptualized the survey design. CB, LI, TA, SR-R, DS, and AB developed the survey questionnaire. CB conceptualized the study design, aims and interpreted the data. CB, AC-G, and TA performed the statistical analysis. CB and LI drafted the first version of the manuscript. AC-G, TA, SR-R, DS, and AB revised the manuscript. All authors read and approved the final manuscript.

## Conflict of Interest

The authors declare that the research was conducted in the absence of any commercial or financial relationships that could be construed as a potential conflict of interest.

## Publisher's Note

All claims expressed in this article are solely those of the authors and do not necessarily represent those of their affiliated organizations, or those of the publisher, the editors and the reviewers. Any product that may be evaluated in this article, or claim that may be made by its manufacturer, is not guaranteed or endorsed by the publisher.

## Abbreviations

SES: socioeconomic status.
