# Supplementary material for: Factors Associated With Dietary Quality During Initial and Later Stages of the COVID-19 Pandemic in Mexico
Source: Front Nutr. 2021 Dec 15;8:758661. doi: 10.3389/fnut.2021.758661 (PMC8714658; doi:10.3389/fnut.2021.758661)
Supplement: Supplementary file 1 [file Table_1.DOCX]

***Supplemental Material***

**Development of the short instrument and performance of the diet quality score.**

The format of the instrument was based on the Nova screener, a short dietary instrument to evaluate the consumption of ultra-processed foods (1,2). However, for our instrument a more comprehensive list of foods and beverages (not limited to ultra-processed foods) was included. Several resources were consulted to inform the selection of the food items, including the Minimum Dietary Diversity for Women (MDD-W) (3), the Prime Diet Quality Score (PDQS)(4), and previously documented food items with high contribution to added sugar and saturated fat intake among the Mexican population (5,6) (**Supplemental Table 1**).

With this short instrument, we estimated a diet quality score. The goal of the score was to capture micronutrient adequacy and key nutrients for chronic diseases such as fiber, saturated fat, and added sugars. Three points were given for the intake of each healthy item or for the non-intake of each unhealthy item. However, to round to 100 total points (50 from eating healthy items, and 50 from not eating unhealthy items) and to increase the score’s ability to predict the target nutrients some food items were given different points as we will explain below.

To refine the scoring system and to evaluate the performance of the score, we used 24-hour dietary recall data from the Mexican National Health and Nutrition Surveys (ENSANUT) 2012 and 2016 (7,8). We included men and non-pregnant non-lactating women ≥18 years old (n=5200). Foods and beverages reported in the 24-hour dietary recall were categorized in the 31 items included in the short dietary instrument. A categorical variable was created to specify if the food item was consumed vs. not consumed. Using the Food Composition Table compiled by the National Institute of Public Health (Base de Alimentos Mexicanos, BAM versión 1.1) (9) we estimated the total intake of calcium, zinc, iron, folate, vitamin A, and vitamin B12, fiber and saturated fat. For added sugar, we used a previous estimation by Sánchez-Pimienta et. al.(5).

Adequate intake of calcium, folate, vitamin A, vitamin B12 and zinc was defined as those above the estimated average requirement (EAR) (10). Adequate intake of iron was defined with the full-probability approach based on the distribution of requirements assuming a 10% bioavailability in the Mexican population (11). Those with a probability of adequacy >50% were considered with adequate intake of iron. A summary measure of overall nutrient adequacy was the total number of nutrients with adequate intake (range 0 to 6).

We ran partial correlations between each of our nutrients of interest [nutrient adequacy, fiber (g/kcal), saturated fat (% kcal), and added sugar (% kcal)] and the categorical variables (consumption yes/no) of the 31 food items of the short instrument simultaneously (e.g., food items were mutually adjusted) (**Supplemental Table 2**). We do not present the partial correlations if the food item contribution to the nutrient was not of interest (e.g., sodas and fiber). Based on these correlations we refined the diet quality scoring. For instance, beef or pork, and milk, cheese or yogurt were given 0 points because although these were highly correlated with saturated fat, they were also correlated with nutrient adequacy. Eggs, sweet bread or cupcakes, desserts or sweets, and potato, wheat or corn tortilla chips were given 5 points because these food items had strong correlations with at least one of the nutrients of interest, and regular soda was given 8 points because its correlation with added sugar was considerably stronger.

Finally, we compared the ability of the diet quality score to predict the nutrients of interest in relation to other well-known diet metrics or indicators(3,6,12), all estimated with the 24-hour recall data (**Supplemental Table 3)**. We found that our short instrument was comparable.

**References**

1. dos Santos Costa C, Rocha de Faria F, Tiemann Gabe K, Fleury Sattamini I, Khandpur N, Helena Marrocos Leite F, Martínez Steele E, Laura da Costa Louzada M, Bertazzi Levy R, Augusto Monteiro C. Nova score for the consumption of ultra-processed foods: description and performance evaluation in Brazil. *Rev Saude Publica* (2021) **55**:13. doi:10.11606/s1518-8787.2021055003588

2. Steele EM, Rauber F, Costa CDS, Leite MA, Gabe KT, Louzada ML da C, Levy RB, Monteiro CA. Dietary changes in the NutriNet Brasil cohort during the covid-19 pandemic. *Rev Saude Publica* (2020) **54**:91. doi:10.11606/s1518-8787.2020054002950

3. Women’s Dietary Diversity Project (WDDP) Study Group. Development of a Dichotomous Indicator for Population-Level Assessment of Dietary Diversity in Women of Reproductive Age. *Curr Dev Nutr* (2017) **1**: doi:10.3945/cdn.117.001701

4. Fung TT, Isanaka S, Hu FB, Willett WC. International food group-based diet quality and risk of coronary heart disease in men and women. *Am J Clin Nutr* (2018) **107**:120–129. doi:10.1093/ajcn/nqx015

5. Sánchez-Pimienta TG, Batis C, Lutter CK, Rivera JA. Sugar-Sweetened Beverages Are the Main Sources of Added Sugar Intake in the Mexican Population. *J Nutr* (2016) **146**:1888–1896.

6. Marrón-Ponce JA, Flores M, Cediel G, Monteiro CA, Batis C. Associations between Consumption of Ultra-Processed Foods and Intake of Nutrients Related to Chronic Non-Communicable Diseases in Mexico. *J Acad Nutr Diet* (2019) **119**:1852–1865. doi:10.1016/j.jand.2019.04.020

7. Lopez-Olmedo N, Carriquiry AL, Rodriguez-Ramirez S, Ramirez-Silva I, Espinosa-montero J, Hernandez-Barrera L, Campirano F, Martinez-Tapia B, Rivera JA, Population M, et al. Usual Intake of Added Sugars and Saturated Fats Is High while Dietary Fiber Is Low in the Mexican Population. *J Nutr* (2016) **146**:1856S-1865S. doi:10.3945/jn.115.218214

8. Ramírez-Silva I, Rodríguez-Ramírez S, Barragán-Vázquez S, Castellanos-Gutiérrez A, Reyes-García A, Martínez-Piña A, Pedroza-Tobías A. Prevalence of inadequate intake of vitamins and minerals in the Mexican population correcting by nutrient retention factors, Ensanut 2016. *Salud Publica Mex* (2020) **62**: doi:10.21149/1109

9. Ramírez Silva I, Rivera Dommarco JA, Barragán Vázquez del Mercado S, Barquera Cervera S, Tolentino Mayo L, Rodríguez Ramírez S, Mejía Rodríguez F, Santos Luna R, Román Pérez S, Grupo BAM. Base de Alimentos de México (BAM): Compilación de la composición de los alimentos frecuentemente consumidos en el país. Versión 1.1. (2019) Available at: www.redpidieta.mx [Accessed May 22, 2019]

10. Institute of Medicine. *Dietary Reference Intakes: The Essential Guide to Nutrient Requirements Dietary Reference Intakes DRI*. Washington DC: National Academies Press (2006).

11. Institute of Medicine (US) Subcommittee on Interpretation and Uses of Dietary Reference Intakes, Institute of Medicine (US) Standing Committee on the Scientific Evaluation of Dietary Reference Intakes. *DRI Dietary Reference Intakes : Applications in Dietary Assessment*. Washington, D.C.: National Academies Press (2000). doi:10.17226/9956

12. Chiuve SE, Fung TT, Rimm EB, Hu FB, McCullough ML, Wang M, Stampfer MJ, Willett WC. *Alternative dietary indices both strongly predict risk of chronic disease*. (2012). doi:10.3945/jn.111.157222

| **Supplemental Table 1. Short diet quality instrument.** |
| --- |
| Dietary intake of the previous day: Take a moment to recall all the foods and beverages that you consumed YESTERDAY, from the moment you woke-up until you went to sleep. |
| **VEGETABLES. Read the options listed below and check everything you ate yesterday:** |
| [ ] green leafy vegetables such as chard, spinach, watercress, purslane, or lettuce |
| [ ] carrot, red or orange pepper |
| [ ] tomato, onion, zucchini, squash, or green beans |
| [ ] any other vegetables such as cucumber, broccoli, beetroot, cactus, etc. |
| [ ] I didn't eat any vegetables |
| **FRUITS. Read the options listed below and check everything you ate yesterday:** |
| [ ] citrus fruits such as orange, lime, tangerine, or grapefruit |
| [ ] cantaloupe, mango, papaya, peach, or mamey sapote |
| [ ] banana or apple |
| [ ] any other fruit such as pineapple, watermelon, strawberries, grapes, guava, etc. |
| [ ] I didn't eat any fruit |
| **PROTEINS. Read the options listed below and check everything that ate yesterday:** |
| [ ] beans, chickpeas, lentils, or fava beans |
| [ ] peanuts, nuts, almonds, chia, sunflower or pumpkin seeds |
| [ ] chicken, fish or seafood |
| [ ] beef or pork |
| [ ] sausage, ham, salami, bacon, or chorizo |
| [ ] eggs |
| [ ] milk, cheese or yogurt |
| [ ] I didn't eat any of the foods listed above |
| **GRAINS. Read the options listed below and check everything you ate yesterday:** |
| [ ] corn tortilla |
| [ ] whole-grain pasta, brown rice, or whole-grain bread |
| [ ] potato, white rice, pasta, white bread, or flour tortilla |
| [ ] oats, wheat bran, wheat germ, or amaranth |
| [ ] I didn't eat any of the foods listed above |
| **SWEET FOODS. Read the options listed below and check everything you ate yesterday:** |
| [ ] sweet bread or cupcakes |
| [ ] cookies |
| [ ] ready-to-eat cereals such as [name of common brands], etc. |
| [ ] desserts or sweets like ice cream, cake, pie, chocolate, marshmallow, gelatin, flan, etc. |
| [ ] I didn't eat any of the foods listed above |
| **SNACKS AND PREPARED FOOD. Read the options listed below and check everything you ate yesterday:** |
| [ ] chips such as [name of common brands], other brand, or generic |
| [ ] ready-to-eat frozen food or instant/canned soups such as pizza, chicken wings, nuggets, [name of common brands] soups |
| [ ] fast food such as [name of common brands], etc. |
| [ ] traditional Mexican fried street food such as tacos, tortas, flautas, chilaquiles, quesadillas, etc. |
| [ ] I didn't eat any of the foods listed above |
| **BEVERAGES. Read the options listed below and check everything you ate yesterday:** |
| [ ] regular soda such as [name of common brands], etc. |
| [ ] diet soda or other diet beverages such as [name of common brands], etc. |
| [ ] bottled fruit juice such as [name of common brands], etc. |
| [ ] flavored beverages such as [name of common brands], etc. |
| [ ] I didn't eat any of the beverages listed above |

| **Supplemental Table 2. Partial correlations between food items and nutrients and diet quality scoring.** | | | | | | | | |
| --- | --- | --- | --- | --- | --- | --- | --- | --- |
|  |  | Partial correlations, *r* | | | |  | Diet Quality Scoring | |
| # | Food item | Nutrient  Adequacy^1^,  n | Fiber,  g/kcal | Saturated  fat,  % kcal | Added  sugar,  % kcal |  | Consumption of healthy foods | Not  consumption of unhealthy foods |
| 1 | green leafy vegetables | 0.05 | 0.02 |  |  |  | 3 |  |
| 2 | red or orange vegetables | 0.09 | 0.04 |  |  |  | 3 |  |
| 3 | tomato, onion, zucchini, squash, or green beans | 0.09 | 0.11 |  |  |  | 3 |  |
| 4 | any other vegetables | 0.05 | 0.09 |  |  |  | 3 |  |
| 5 | citrus fruits | 0.04 | 0.24 |  |  |  | 3 |  |
| 6 | red or orange fruits | 0.06 | 0.06 |  |  |  | 3 |  |
| 7 | banana or apple | NS | 0.10 |  |  |  | 3 |  |
| 8 | any other fruit. | 0.03 | 0.06 |  |  |  | 3 |  |
| 9 | legumes | 0.08 | 0.32 | -0.19 |  |  | 3 |  |
| 10 | nuts and seeds | 0.04 |  | -0.04 |  |  | 3 |  |
| 11 | chicken, fish or seafood | 0.02 |  | -0.04 |  |  | 3 |  |
| 12 | beef or pork | **0.16** |  | **0.20** |  |  | 0 | 0 |
| 13 | processed meat | 0.07 |  | 0.10 |  |  |  | 3 |
| 14 | eggs | **0.10** |  | NS |  |  | 5 |  |
| 15 | milk, cheese or yogurt | **0.16** |  | **0.30** |  |  | 0 | 0 |
| 16 | corn tortilla | 0.02 | 0.16 | -0.17 | -0.06 |  | 3 |  |
| 17 | whole-grain pasta, rice or bread | 0.02 | 0.02 | NS | 0.03 |  | 3 |  |
| 18 | potato, refined rice, pasta, bread, or flour tortilla | NS | -0.06 | -0.10 | NS |  | 3 |  |
| 19 | oats, wheat bran, wheat germ, or amaranth | 0.04 | 0.06 | -0.05 | 0.02 |  | 3 |  |
| 20 | sweet bread or cupcakes |  | -0.06 | **0.18** | 0.06 |  |  | 5 |
| 21 | cookies |  | -0.07 | NS | 0.09 |  |  | 3 |
| 22 | ready-to-eat cereals |  |  | -0.06 | 0.08 |  |  | 3 |
| 23 | desserts or sweets |  | -0.07 | 0.07 | **0.22** |  |  | 5 |
| 24 | potato, wheat or corn tortilla chips |  | -0.07 | **0.12** | -0.05 |  |  | 5 |
| 25 | ready-to-eat frozen food or instant/canned soups |  | -0.02 | NS | 0.02 |  |  | 3 |
| 26 | fast food from chains |  | -0.05 | 0.04 | 0.03 |  |  | 3 |
| 27 | traditional Mexican fried street food |  | NS | NS | 0.02 |  |  | 3 |
| 28 | regular soda |  | -0.11 | -0.11 | **0.44** |  |  | 8 |
| 29 | diet soda or other diet beverages |  | NS | NS | 0.02 |  |  | 3 |
| 30 | bottled fruit juice |  | -0.08 | -0.04 | NS |  |  | 3 |
| 31 | flavored beverages |  | -0.02 | -0.02 | 0.08 |  |  | 3 |
|  | Total score |  |  |  |  |  | 50 | 50 |
| NS: Not Significant, partial correlation had a p-value >0.05.  Bold numbers: strong correlations that motivated the different scoring.  ^1^Number of nutrients (calcium, zinc, iron, folate, vitamin A, and vitamin B12) with adequate intake. | | | | | | | | |

| **Supplemental Table 3. Correlation between diet quality scores and indicators with nutrients of interest.** | | | | |
| --- | --- | --- | --- | --- |
|  | Correlations^1^, *r* | | | |
|  | Nutrient  Adequacy^2^, n | Fiber,  g/kcal | Saturated fat,  % kcal | Added sugar,  % kcal |
| COVID-19 online survey diet quality score | 0.07 | 0.36 | -0.24 | -0.32 |
| Alternate healthy eating index (AHEI-2010) | -0.14 | 0.42 | -0.28 | -0.35 |
| Minimum dietary diversity for women (MDD-W) | 0.29 | 0.13 | -0.04 | 0.17 |
| Energy share of ultra-processed foods | 0.02 | -0.43 | 0.29 | 0.31 |
| ^1^All correlations are p<0.05  ^2^Number of nutrients (calcium, zinc, iron, folate, vitamin A, and vitamin B12) with adequate intake. | | | | |

| **Supplemental Table 4. Mean diet quality score by sociodemographic and individual characteristics during 1^st^ survey round (n=3,131)** | | |
| --- | --- | --- |
|  | Diet quality score; mean (95% CI) | p-value vs. reference |
| Sex, |  |  |
| Female | 64.6 (64.2, 65) | ref |
| Male | 62.8 (62, 63.5) | <0.001 |
| Age, % |  |  |
| 18-30 y | 62.7 (62, 63.5) | ref |
| 31-40 y | 63.2 (62.5, 63.9) | 0.339 |
| 41-50 y | 64.3 (63.5, 65.1) | 0.005 |
| 51-60 y | 66.2 (65.2, 67.2) | <0.001 |
| >60 y | 67.4 (66.2, 68.5) | <0.001 |
| Marital Status |  |  |
| Single | 63.4 (62.8, 64) | ref |
| Married or with partner | 64.5 (64, 65) | 0.009 |
| Divorced/separated/widowed | 64.7 (63.5, 66) | 0.054 |
| Head of the household highest education level |  |  |
| Secondary school or less | 61.6 (60.3, 63) | ref |
| High school | 62 (60.9, 63.1) | 0.690 |
| Bachelor degree | 63.8 (63.2, 64.4) | 0.004 |
| Graduate degree | 65.6 (65, 66.2) | <0.001 |
| Main occupation before the pandemic |  |  |
| Student or working | 63.8 (63.4, 64.3) | ref |
| Other | 65 (64.2, 65.7) | 0.008 |
| Socieconomic status |  |  |
| High (A/B) | 63.5 (62.7, 64.4) | ref |
| Middle high (C+) | 65 (64.4, 65.6) | 0.006 |
| Middle low (C and C-) | 63.9 (63.3, 64.5) | 0.527 |
| Low (D+ and D) | 61.9 (60.1, 63.6) | 0.093 |
| Beneficiary of social programs |  |  |
| None | 64.1 (63.7, 64.5) | ref |
| Financial aid | 66.9 (64.9, 68.9) | 0.006 |
| Other | 61.4 (59.2, 63.6) | 0.019 |
| Geographical region |  |  |
| South | 63.6 (62.7, 64.5) | ref |
| Center | 63.9 (63.1, 64.7) | 0.592 |
| North | 64 (62.9, 65.1) | 0.550 |
| Mexico City Metropolitan Area | 64.7 (64.1, 65.2) | 0.050 |
| Guadalajara Metropolitan Area | 63.6 (62.3, 64.9) | 0.985 |
| Municipality population size |  |  |
| ≥100,000 habs. | 64.2 (63.8, 64.5) | ref |
| <100,000 habs. | 63.9 (62.7, 65.2) | 0.752 |
| Household with children (<18 y) |  |  |
| No | 64.6 (64.1, 65) | ref |
| Yes | 63.5 (62.9, 64.1) | 0.007 |
| Healthy food consiousness |  |  |
| Always | 68.9 (68.2, 69.5) | ref |
| Almost always | 64.4 (63.9, 64.9) | <0.001 |
| Sometimes or never | 56.5 (55.7, 57.3) | <0.001 |

| **Supplemental Table 5. Association between pandemic related-factors and diet quality score during the 2^nd^ survey round (n=1,703)^1^** | | |
| --- | --- | --- |
|  | Model 1 | Model 2 |
| Food prepared away-from-home the day before |  |  |
| None | 0 (ref) | 0 (ref) |
| Restaurant (includes take-out and delivery) | **-3.9 (-5.1, -2.7)** | **-3.2 (-4.4, -2.1)** |
| Street vendors | **-5.4 (-7.8, -3.1)** | **-4.3 (-6.5, -2)** |
| Traditional or street market purchases, now |  |  |
| in-store | 0 (ref) | 0 (ref) |
| in-store and home delivery | 0.1 (-4, 4.1) | -0.7 (-4.4, 3.1) |
| home delivery | 1.1 (-0.7, 2.8) | 1 (-0.7, 2.8) |
| none | -0.7 (-1.7, 0.4) | **-1.3 (-2.3, -0.2)** |
| Grocery store purchases, now |  |  |
| in-store | 0 (ref) | 0 (ref) |
| in-store and home delivery | 0.3 (-1.9, 2.4) | 0.3 (-1.7, 2.3) |
| home delivery | -0.1 (-1.5, 1.4) | -0.2 (-1.7, 1.2) |
| none | -1 (-2.3, 0.3) | **-1.5 (-2.8, -0.3)** |
| Level of home confinement |  |  |
| going out for motives other than work | 0 (ref) | 0 (ref) |
| not leaving the home | 0.3 (-2.2, 2.8) | 0.8 (-1.6, 3.1) |
| going out to work ≤3 times/week | 0.3 (-1, 1.7) | 0.6 (-0.7, 1.9) |
| going out to work ≥4 times/week | **-2.6 (-3.8, -1.3)** | **-1.7 (-2.9, -0.4)** |
| Income changes |  |  |
| Without change | 0 (ref) | 0 (ref) |
| Increased | **-3.9 (-6.1, -1.6)** | **-3 (-5.1, -0.8)** |
| Decreased somewhat | 0 (-1.2, 1.1) | 0.2 (-1, 1.3) |
| Decreased a lot | 0.6 (-0.7, 1.9) | 1.2 (-0.1, 2.5) |
| Perceived change in free time |  |  |
| Without change | 0 (ref) | 0 (ref) |
| Decreased | -0.6 (-1.8, 0.6) | 0.2 (-1, 1.4) |
| Increased | -0.6 (-1.9, 0.6) | -0.9 (-2.1, 0.3) |
| Perceived change in time for cooking |  |  |
| Without change | 0 (ref) | 0 (ref) |
| Decreased | **-5.4 (-7.5, -3.2)** | **-3.2 (-5.3, -1)** |
| Increased | 0 (-1.2, 1.2) | 0.3 (-1, 1.5) |
| Food is prepared by others | **-2.8 (-4.2, -1.3)** | **-2.2 (-3.7, -0.8)** |
| Perceived change in interest in eating healthy |  |  |
| Without change | 0 (ref) | 0 (ref) |
| Decreased | **-4.7 (-6.6, -2.8)** | **-3.2 (-5.1, -1.3)** |
| Increased | -0.4 (-1.5, 0.6) | -0.3 (-1.3, 0.7) |
| Eating more due to anxiety, depression or boredom |  |  |
| No | 0 (ref) | 0 (ref) |
| Yes | **-2 (-3, -1)** | **-1.6 (-2.5, -0.6)** |
| Food insecurity^2^ |  |  |
| Without difficulty | 0 (ref) | 0 (ref) |
| Cheaper foods or that I enjoy less | -1 (-2.5, 0.4) | -1 (-2.4, 0.5) |
| Skip meals, eat less, or do not eat in an entire day | **-3.7 (-5.9, -1.5)** | **-2.6 (-4.7, -0.6)** |
| Stockpiling food |  |  |
| None | 0 (ref) | 0 (ref) |
| Only basic foods | -0.6 (-2, 0.7) | -0.4 (-1.6, 0.9) |
| Junk food | **-4.8 (-7.4, -2.1)** | **-4.3 (-6.7, -1.9)** |
| Restriction level |  |  |
| Green | 0 (ref) | 0 (ref) |
| Yellow | -1.5 (-5.1, 2.1) | -0.7 (-4.3, 2.9) |
| Orange | -2.2 (-5.8, 1.4) | -1.2 (-4.8, 2.5) |
| Red | -3.8 (-8.8, 1.3) | -3.1 (-8.0, 1.8) |
| ^1^Weighted to maintain the distribution of sociodemographic variables of 1^st^ round’s participants. ^2^Dificulty eating enough due to economic constraints. Model 1 adjusted by sex, age category, marital status, education level from head of household, main occupation before the pandemic, SES, beneficiary of social programs, geographic region, municipality population size, household with children, and healthy food consciousness. Model 2 adjusted by covariates from Model 2, plus all the other pandemic-factors listed in this table. | | |

| **Supplemental Table 6. Distribution of pandemic related factors during 1st (Jun-Jul 2020) and 2nd (Nov-Dec 2020) survey round in Mexico, among subjects that participated in both surveys.** | | | | | | | | |
| --- | --- | --- | --- | --- | --- | --- | --- | --- |
|  | 1^st^ round | 2^nd^ round |  | 1^st^ round | | 2^nd^ round | | |
|  | All participants  (n=3,131) | Reported participating in 1^st^ round (n=766)^1^ |  | Confirmed participation in both rounds (n=552)^1^ | | | | |
|  | p=0.112 | |  | p=0.643 | | | | |
| **Diet quality score, mean** | 64.1 (63.8, 64.5) | 64.9 (64.1, 65.7) |  | 65.7 (64.8, 66.6) | | | | 65.4 (64.4, 66.4) |
| **Perceived changes in diet, physical activity and body weight** |  |  |  |  | | | |  |
| Perceived change in intake of healthy food | p=0.205 | |  | p=0.363 | | | | |
| Without change | 46 | 48 |  | 45 | | | | 46 |
| Decreased | 12 | 13 |  | 11 | | | | 13 |
| Increased | 42 | 38 |  | 44 | | | | 41 |
| Perceived change in intake of junk food | p<0.001 | |  | p=0.070 | | | | |
| Without change | 38 | 35 |  | 38 | | | | 35 |
| Decreased | 39 | 48 |  | 42 | | | | 49 |
| Increased | 23 | 17 |  | 20 | | | | 16 |
| Perceived change in physical activity | p=0.180 | |  | p=0.022 | | | | |
| Without change | 23 | 26 |  | 18 | | | | 25 |
| Decreased | 53 | 50 |  | 52 | | | | 51 |
| Increased | 24 | 25 |  | 30 | | | | 24 |
| Perceived change in body weight | p=0.003 | |  | p=0.571 | | | | |
| Without change | 39 | 35 |  | 40 | | | | 36 |
| Decreased | 25 | 32 |  | 27 | | | | 31 |
| Increased | 36 | 33 |  | 33 | | | | 33 |
| **Pandemic related factors, %** |  |  |  |  | | | | |
| Food prepared away-from-home the day before | p<0.001 | |  | p<0.001 | | | | |
| None | 82 | 72 |  | 85 | | 73 | | |
| Restaurant (includes take-out and delivery) | 15 | 23 |  | 12 | | 21 | | |
| Street vendors | 3 | 5 |  | 3 | | 6 | | |
| Traditional or street market purchases, before | p=0.289 | |  | p=0.631 | | | | |
| in-store | 63 | 66 |  | 67 | | | 67 | |
| in-store and home delivery | 1 | 1 |  | 2 | | | 1 | |
| home delivery | 2 | 1 |  | 1 | | | 2 | |
| none | 34 | 32 |  | 31 | | | 30 | |
| Traditional or street market purchases, now | p<0.001 | |  | p=0.049 | | | | |
| in-store | 33 | 42 |  | 33 | | 42 | | |
| in-store and home delivery | 2 | 2 |  | 3 | | 2 | | |
| home delivery | 13 | 11 |  | 13 | | 11 | | |
| none | 52 | 45 |  | 51 | | 45 | | |
| Traditional or street market purchases, before | p=0.316 | |  | p=0.854 | | | | |
| in-store | 80 | 77 |  | 79 | 77 | | | |
| in-store and home delivery | 3 | 3 |  | 2 | 3 | | | |
| home delivery | 2 | 2 |  | 3 | 3 | | | |
| none | 15 | 17 |  | 17 | 17 | | | |
| Grocery store purchases, now | p=0.001 | |  | p=0.463 | | | | |
| in-store | 59 | 62 |  | 60 | | 62 | | |
| in-store and home delivery | 8 | 5 |  | 8 | | 5 | | |
| home delivery | 17 | 13 |  | 15 | | 14 | | |
| none | 16 | 19 |  | 17 | | 18 | | |
| Level of home confinement | p<0.001 | |  | p<0.001 | | | | |
| going out for motives other than work | 61 | 57 |  | 65 | | 56 | | |
| not leaving the home | 7 | 4 |  | 5 | | 4 | | |
| going out to work ≤3 times/week | 17 | 17 |  | 16 | | 16 | | |
| going out to work ≥4 times/week | 15 | 23 |  | 14 | | 25 | | |
| Income changes | p<0.001 | |  | p=0.155 | | | | |
| Without change | 49 | 50 |  | 51 | | 49 | | |
| Increased | 2 | 6 |  | 3 | | 5 | | |
| Decreased somewhat | 26 | 23 |  | 24 | | 25 | | |
| Decreased a lot | 23 | 21 |  | 23 | | 21 | | |
| Perceived change in free time | p<0.001 | |  | p=0.001 | | | | |
| Without change | 20 | 25 |  | 20 | | 26 | | |
| Decreased | 27 | 36 |  | 29 | | 35 | | |
| Increased | 52 | 39 |  | 51 | | 39 | | |
| Perceived change in time for cooking | p<0.001 | |  | p=0.008 | | | | |
| Without change | 19 | 22 |  | 20 | | 23 | | |
| Decreased | 4 | 7 |  | 3 | | 7 | | |
| Increased | 52 | 44 |  | 52 | | 45 | | |
| Food is prepared by others | 25 | 26 |  | 25 | | 25 | | |
| Perceived change in interest in eating healthy | p=0.761 | |  | p=0.187 | | | | |
| Without change | 41 | 40 |  | 42 | | 37 | | |
| Decreased | 7 | 7 |  | 5 | | 6 | | |
| Increased | 52 | 53 |  | 53 | | 57 | | |
| Eating more due to anxiety, depression or boredom | p<0.001 | |  | p=0.083 | | | | |
| No | 53 | 60 |  | 54 | | 59 | | |
| Yes | 47 | 40 |  | 46 | | 41 | | |
| Food insecurity^2^ | p=0.016 | |  | p=0.506 | | | | |
| Without difficulty | 79 | 84 |  | 77 | | 80 | | |
| Cheaper foods or that I enjoy less | 14 | 12 |  | 18 | | 15 | | |
| Skip meals, eat less, or do not eat in an entire day | 7 | 4 |  | 5 | | 4 | | |
| Stockpiling food | p<0.001 | |  | p<0.001 | | | | |
| None | 61 | 87 |  | 61 | | 89 | | |
| Only basic foods | 33 | 10 |  | 33 | | 8 | | |
| Junk food | 7 | 3 |  | 5 | | 3 | | |
| Level of restriction | p<0.001 | |  | p<0.001 | | | | |
| Green | 0 | 1 |  | 0 | | 2 | | |
| Yellow | 0 | 17 |  | 0 | | 17 | | |
| Orange | 55 | 79 |  | 59 | | 79 | | |
| Red | 45 | 3 |  | 41 | | 2 | | |
| ^1^Weighted to maintain the distribution of sociodemographic variables of all (n=3,131) first round’s participants.  ^2^Dificulty eating enough due to economic constraints | | | | | | | | |
